# Rare Cutaneous Metastasis From Esophageal Squamous Cell Carcinoma

**DOI:** 10.1002/kjm2.70249

**Published:** 2026-06-22

**Authors:** Sarah Hsin Cheng, Yen‐Shuo Huang, Chi‐En Hsiao, Hui‐Hua Hsiao

**Affiliations:** ^1^ Department of Clinical Education and Training, Kaohsiung Medical University Hospital Kaohsiung Medical University Kaohsiung Taiwan; ^2^ Department of Pathology, Kaohsiung Medical University Hospital Kaohsiung Medical University Kaohsiung Taiwan; ^3^ Department of Molecular and Cell Biology University of California Berkeley California USA; ^4^ Cancer Center and Internal Medicine Kaohsiung Medical University Hospital Kaohsiung Taiwan; ^5^ Faculty of Medicine Kaohsiung Medical University Kaohsiung Taiwan

Although the prevalence of esophageal cancer is not high, it remains one of the cancers with the worst prognosis, with a 5‐year survival rate of approximately 22% [1]. There are two major histological types: squamous cell carcinoma and adenocarcinoma. Both types rarely metastasize to the skin. Here, we present a patient with esophageal squamous cell carcinoma who developed a rare cutaneous metastasis during therapy.

An indurated erythematous nodule on the left upper back of a 57‐year‐old male esophageal cancer patient was discovered by physical examination in his fifth course of chemotherapy in December 2024. He was diagnosed with upper‐third esophageal squamous cell carcinoma, grade 3, with liver metastasis (T4bN3M1, stage IVB), and received PF (cisplatin 75 mg/m^2^ + 5‐FU [5‐fluorouracil] 1000 mg/m^2^ × 5 days) chemotherapy with stable disease. The skin lesion present on the shoulder consisted of painless nodules without discharge or ulceration (Figure 1A). Due to its nature, a skin biopsy was performed. The pathology revealed keratinizing squamous cell carcinoma infiltrating the dermis without dysplastic changes in the overlying epidermis. Lymphovascular invasion by tumor cells was also observed (Figure 1B,C), supporting the diagnosis of cutaneous metastasis from esophageal squamous cell carcinoma.

**FIGURE 1:**
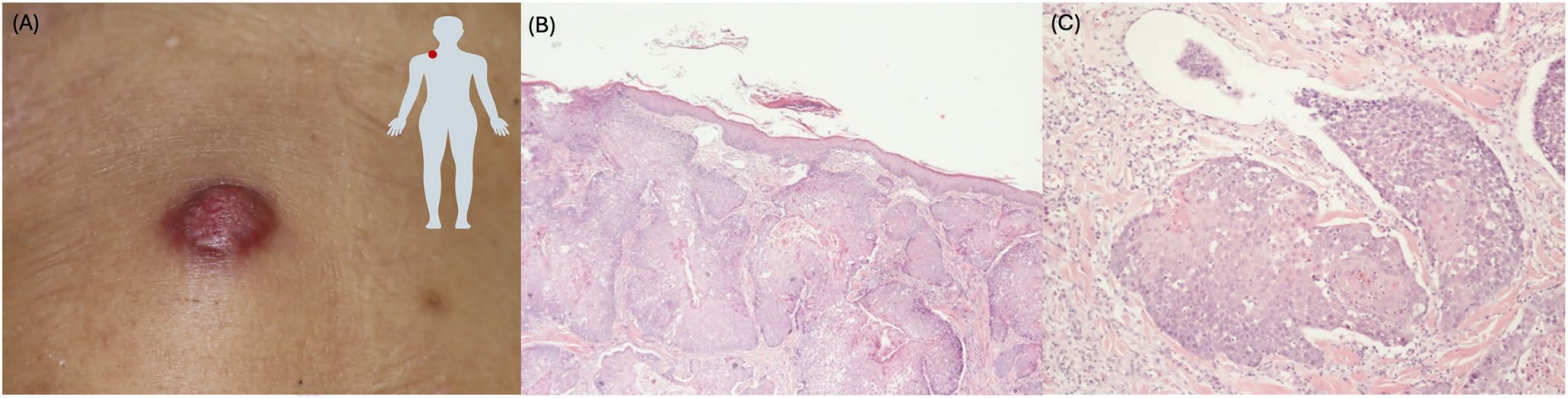
(A) A solitary, indurated erythematous nodule was observed on the patient's left upper back, present for an unknown duration. On the right upper corner is a schematic diagram showing the location of the nodule from the posterior view of the patient. (B) Histological examination revealed keratinizing squamous cell carcinoma infiltrating the dermis, without dysplastic changes in the overlying epidermis (hematoxylin and eosin stain; original magnification ×40). (C) At higher magnification, invasive nests of large, irregular neoplastic cells with keratinization and evidence of lymphovascular invasion are seen in the dermis (hematoxylin and eosin stain; original magnification ×100).

Shortly after the diagnosis, the patient was admitted to our emergency department with symptoms including severe headache, nausea, vomiting, and altered consciousness. Imaging studies revealed multiple metastases, including to the brain. After being informed of the poor prognosis, the patient and his family chose hospice care. He passed away from cancer‐associated multi‐organ failure 1 month later.

Esophageal cancer is among the most lethal malignancies. According to recent statistics from the United States, its 5‐year survival rate is approximately 22%, comparable to lung cancer (27%) and pancreatic cancer (13%) [1]. The two major histological subtypes are squamous cell carcinoma and adenocarcinoma, both of which rarely metastasize to the skin [2, 3]. We report a case of cutaneous metastasis from esophageal squamous cell carcinoma, which is even less common than that from esophageal adenocarcinoma [2]. Most reported cases present as painless, solitary, or scattered nodules on the head [4]. Involvement of the upper back or shoulder is even less common [4].

The mechanism behind cutaneous metastasis of esophageal cancer remains poorly understood. In addition to tumor–host microenvironment interactions, wound healing and trans‐epidermal elimination have been proposed as possible mechanisms [4, 5]. In our case, the initial differential diagnosis before biopsy included cyst, local skin infection, or cutaneous malignancy. This case highlights the importance of considering skin biopsy to rule out cutaneous metastasis, even in cancers that rarely spread to the skin, such as esophageal squamous cell carcinoma.

## Conflicts of Interest

The authors declare no conflicts of interest.

## Data Availability

The data that support the findings of this study are available on request from the corresponding author. The data are not publicly available due to privacy or ethical restrictions.
